# Determining Pain Pressure Thresholds and Muscle Stiffness Cut-Offs to Discriminate Latent Myofascial Trigger Points and Asymptomatic Infraspinatus Muscle Locations: A Diagnostic Accuracy Study

**DOI:** 10.3390/diagnostics15202633

**Published:** 2025-10-18

**Authors:** Mateusz D. Kobylarz, Ricardo Ortega-Santiago, Sandra Sánchez-Jorge, Marcin Kołacz, Dariusz Kosson, Germán Monclús-Díez, Juan Antonio Valera-Calero, Mónica López-Redondo

**Affiliations:** 1 Escuela Internacional de Doctorado, Universidad Rey Juan Carlos, 28922 Alcorcon, Spain; 2 Akademia Terapii Manualnej i Igłoterapii Suchej (ATMIS), 34-400 Nowy Targ, Poland; 3 Department of Physical Therapy, Occupational Therapy, Rehabilitation and Physical Medicine, Universidad Rey Juan Carlos, 28922 Alcorcón, Spain; ricardo.ortega@urjc.es; 4 Grupo de Investigación de Alto Rendimiento en Evaluación Multidimensional y Tratamiento del Dolor Crónico, Universidad Rey Juan Carlos, 28922 Alcorcon, Spain; 5 Faculty of Health Sciences, Universidad Francisco de Vitoria, 28223 Madrid, Spain; 6 1st Department of Anaesthesiology and Intensive Care, Medical University of Warsaw, 02-005 Warsaw, Poland; 7 Department of Anaesthesiology and Intensive Care, Division of Teaching, Medical University of Warsaw, 02-005 Warsaw, Poland; 8 Department of Physiotherapy, Faculty of Nursery, Physiotherapy and Podiatry, Complutense University of Madrid, 28040 Madrid, Spain; 9 Grupo InPhysio, Instituto de Investigación Sanitaria del Hospital Clínico San Carlos (IdISSC), 28040 Madrid, Spain

**Keywords:** diagnostic accuracy, myofascial pain syndrome, myofascial trigger points, pain pressure threshold, shear wave elastography, ultrasound imaging

## Abstract

**Background**: Latent myofascial trigger points (MTrPs) are clinically relevant because they lower local pressure pain thresholds (PPTs), can perturb motor control, and may sustain shoulder symptoms even when overt pain is absent. However, even if previous studies assessed stiffness and mechanosensitivity differences between MTrPs and asymptomatic regions, objective patient-level cut-offs and diagnostic-accuracy metrics to distinguish latent MTrPs from adjacent asymptomatic tissue are lacking. **Objective**: To quantify the diagnostic accuracy of pressure algometry (PPT) and shear-wave elastography (SWE) for distinguishing latent MTrPs from adjacent asymptomatic tissue. **Methods**: A single-center cross-sectional study was conducted including 76 volunteers with ≥1 latent infraspinatus MTrP (assessed by following the current Delphi consensus criteria). The most sensitive latent MTrP and a control site 2 cm cranial was measured on the dominant side infraspinatus muscle in each participant. PPT and SWE were acquired with a standardized protocol (long-axis imaging, anisotropy control, minimal probe pressure; three captures per site; 1 cm rectangular ROI; operator blinded to site type). ROC analyses estimated areas under the curve (AUCs), Youden-optimal cut-offs, sensitivity, specificity, and likelihood ratios (LR+/−). **Results**: Latent MTrPs showed lower PPTs than controls (*p* < 0.001) and higher stiffness (shear modulus: *p* = 0.009; shear-wave speed: *p* = 0.022). PPT yielded AUC = 0.704 with an optimal cut-off of 47.5 N (sensitivity 0.75; specificity 0.592; LR+ 1.84; LR− 0.42), outperforming SWE metrics (shear modulus AUC 0.611; cut-off 23.6 kPa; sensitivity 0.632; specificity 0.605; LR+ 1.60; LR− 0.61; shear-wave speed AUC 0.601; cut-off 2.55 m/s; sensitivity 0.592; specificity 0.632; LR+ 1.61; LR− 0.65). **Conclusions**: In the infraspinatus, PPT provides moderate discrimination between latent MTrPs and adjacent asymptomatic tissue, whereas resting SWE—despite small mean differences—exhibited lower accuracy. These findings support mechanosensitivity as a primary measurable signal and position SWE as an adjunct. External validation across devices and operators, and multivariable models integrating sensory, imaging, and clinical features, are warranted.

## 1. Introduction

Myofascial pain syndrome (MPS) is a regional musculoskeletal condition in which pain and dysfunction arise from myofascial trigger points (MTrPs, which are defined as hyper-irritable foci embedded in palpable taut bands of muscle) [1,2,3]. On examination, these MTrP loci are distinctly hypersensitive and can reproduce the patient’s symptoms and provoke non-dermatomal referred pain; motor impairments and occasional autonomic signs may accompany the sensory picture [4]. Clinically, MTrPs form a spectrum with two phenotypic expressions (active and latent) that share a common substrate yet differ in how they manifest to patients and examiners [5,6].

Active MTrPs are those for which mechanical stimulation (typically flat or pincer palpation within a taut band) elicits the patient’s familiar, recognizable symptoms. This reproduction may include the same quality and distribution of pain for which the patient sought care, along with the characteristic, non-dermatomal spread from a discrete muscular source. Patients with active points frequently report spontaneous pain at rest or during ordinary movements, and they often demonstrate measurable functional deficits such as reduced range of motion, pain-inhibited weakness, and movement avoidance. In some regions, activation can provoke autonomic accompaniments (e.g., mild vasomotor or sudomotor changes) that further differentiate the clinical picture. From a pathophysiological standpoint, active points are thought to generate a more intense and sustained nociceptive barrage, which can contribute to segmental hyperexcitability and features of central sensitization, helping to explain disproportionate pain intensity and after-sensations in certain individuals [7,8,9].

On the other hand, latent MTrPs occupy the same anatomical niche (a tender, hypersensitive spot within a taut band) but do not reproduce symptoms that the patient recognizes as their usual complaint when palpated [9]. At rest they are typically asymptomatic or produce only local discomfort when provoked; nevertheless, they are not clinically inert [10]. Latent points can restrict extensibility, alter muscle recruitment timing, diminish strength endurance through reflex inhibition, and degrade motor control, thereby predisposing to overload of adjacent tissues and perpetuation of faulty movement patterns [11,12]. In daily life, they are often unmasked by sustained low-level loading, prolonged posture, or unaccustomed effort, at which point they may transition toward an active presentation if peripheral and central facilitators align [7,8,9].

In practice, in order to improve consistency, the latest update [13] recommends identifying MTrPs using a small cluster of converging signs (presence of a taut band, a hypersensitive spot within that band, and referred pain or sensation when provoked) while recognizing that individual referral patterns vary and should not be treated as rigid, muscle-specific “maps.” Within this framework, the active–latent label becomes a clinically meaningful modifier that signals expected symptom behavior and informs priorities for assessment and follow-up. Since objective and adjunctive assessments can contextualize (but not replace) clinical evaluation, instrumental physical assessments are of interest as complementary procedures during the diagnosis.

For instance, pressure algometry is a quantitative sensory testing method used to objectively assess mechanical pain sensitivity in soft tissues [14]. It measures the pressure pain threshold (PPT), defined as the minimal amount of pressure that elicits a transition from a sensation of pressure to one of pain [15]. Using a handheld algometer, pressure is applied at a constant rate perpendicularly to the skin surface over specific anatomical sites until the subject reports pain onset. The PPT reflects both peripheral and central sensitization phenomena [16]. Owing to short testing time [17], and good intra- and inter-rater reliability [18], pressure algometry has become a widely accepted and reproducible tool for quantifying deep tissue mechanosensitivity in musculoskeletal research and clinical practice [19].

On the other hand, tools like shear-wave elastography (SWE, an ultrasound-based imaging technique that quantifies tissue mechanical properties by measuring the propagation speed of shear waves generated by acoustic radiation force [20]) provide objective measures of muscle stiffness. The velocity of these shear waves is directly related to tissue stiffness: faster propagation indicates greater stiffness, which can be expressed either as shear-wave speed (m/s) or as shear modulus (kPa) through shear modulus conversion [21]. Given that two of the five mandatory diagnostic criteria for MPS (presence of a taut band and tenderness within the MTrP) are directly related to tissue stiffness [13], SWE emerges as a promising tool as provides objective, quantitative, and spatially resolved maps of tissue elasticity (unlike manual palpation [22]), making it particularly useful for characterizing the mechanical environment of MTrPs .

Given the clinical relevance of latent MTrPs [23,24], and acknowledging that several investigations [25,26] have already contrasted PPT and SWE derived stiffness between latent points in the infraspinatus and nearby asymptomatic tissue, the current evidence base still stops short of what clinicians ultimately need: patient-level classification metrics. Although prior work has shown that PPTs are lower over latent MTrPs than over control sites, whereas SWE often fails to demonstrate localized stiffness differences within the infraspinatus [25,26], those studies concentrated on mean contrasts and reliability rather than on test performance indices. Therefore, the aim of this study is to determine the diagnostic accuracy of both pressure algometry and shear wave elastography to discriminate latent MTrPs from asymptomatic regions within the infraspinatus muscle.

## 2. Materials and Methods

### 2.1. Study Design

We carried out a cross-sectional, observational investigation to examine the PPT and muscle stiffness cut-off points for differentiating latent myofascial trigger points (MTrPs) and asymptomatic regions within the infraspinatus muscle. The study was conducted in a physiotherapy clinic and followed STARD recommendations [27] as well as SQUIRE 2.0 guidance [28]. Participant rights were protected in accordance with the Declaration of Helsinki, and a local ethics committee approved the protocol.

### 2.2. Participants

Eligibility required the presence of at least one latent infraspinatus MTrP identified per the most recent Delphi consensus [7,29] (i.e., a palpable taut band within skeletal muscle containing a hypersensitive spot that is tender to palpation and may provoke localized discomfort). Volunteers provided written informed consent prior to enrollment.

Exclusions comprised: age <18 or >65 years; current use of medications that could alter muscle tone; prior shoulder or spine surgery; traumatic injuries; neuropathies; serious medical comorbidities; active MTrPs (those for which mechanical stimulation elicits the patient’s familiar, recognizable symptoms [7]); clinically meaningful left–right asymmetries in posture, structure, or function; and widespread pain disorders such as fibromyalgia.

The minimum sample size required for adequate statistical power was calculated based on the recommendations for diagnostic accuracy studies provided by Hajian-Tilaki [30], which is based on the formula n = Z^2^ × p(1 − p)/d^2^ for estimating a single proportion with a specified confidence interval width. In this formula, Z is the standard normal deviate corresponding to a 95% confidence level (1.96), p is the expected proportion (sensitivity or specificity), and d is the desired half-width of the confidence interval (precision). Considering that a sensitivity of at least 0.75 represents a “good” diagnostic performance [31], an expected sensitivity of 0.75 and a desired precision of ±0.10 were assumed. Substituting these values into the formula, results indicate that 73 participants would be required to estimate sensitivity with 95% confidence and ±10% precision.

### 2.3. Procedures: Determining the Presence of Latent Trigger Points

A first single clinician with >10 years of practice identified and classified MTrPs in all participants. Subjects lay prone with arms alongside the trunk and the shoulder maintained in neutral to limit passive stretch that could influence SWE measurements. The examiner surveyed the dominant infraspinatus using flat palpation and applied the established latent MTrP criteria.

To avoid dependence among observations and inflated precision from correlated bilateral measurements, only one side (the dominant side) was assessed, improving standardization across right- and left-handed individuals.

When multiple latent MTrPs were present, the most symptomatic site was selected and marked with a blue skin pen. A control site was also marked 2 cm cranial to the MTrP on different fibers (in the infraspinatus muscle, fibers run predominantly in a medio-lateral orientation. Therefore, a 2 cm cranially displacement occurs along the longitudinal axis of the fibers, ensuring that the control point corresponded to a different set of fibers and not the same taut band [32]). This 2 cm spacing was chosen (1) in light of prior work demonstrating spontaneous electrical activity within MTrPs (which is absent at nearby control sites ~1 cm away) and significant between-site differences in mechanosensitivity at fixed 1 cm offsets [33] and (2) to minimize the likelihood of mechanical overlap within the same taut band, given the anatomical thickness and fascicular arrangement of the infraspinatus in this region [34]. The examiner verified that this control location did not meet the criteria for a second latent MTrP.

**Figure 1:**
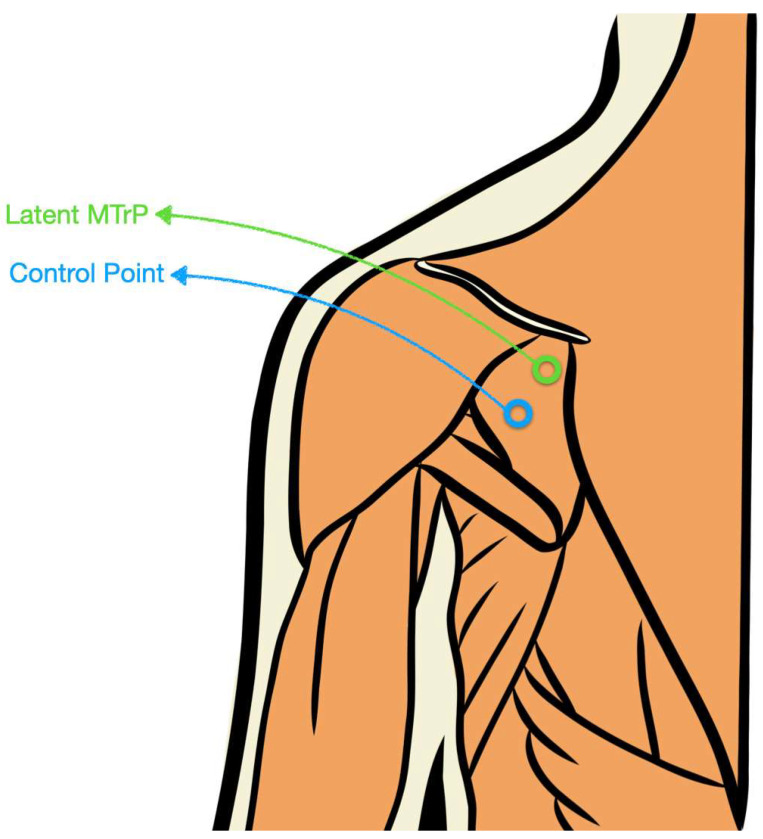
Schematic illustration of the latent MTrP and control point location within the infraspinatus muscle.

Because both marks were identical and the ultrasound operator was not told which was the MTrP versus control (nor the rationale for the locations), effective blinding was achieved. A schematic representation of this procedure is illustrated in Figure 1.

### 2.4. Assessments: Pain Pressure Thresholds

PPTs at the MTrP and control locations were obtained with a digital algometer (Commander™, JTECH Medical Industries Inc., Midvale, UT, USA; 1 cm tip) by the US operator, applying pressure at ~5 N/s. Participants received standardized instructions to say “now” at the first sensation of pain (as opposed to pressure). For each site, the mean of three trials was used in analyses. To minimize bias, a second single operator was blinded to the recorded PPT values; the palpating examiner documented the readings independently without feedback. This procedure showed to be acceptably reliable in both locations (latent MTrPs had an intraclass correlation coefficient ICC = 0.840; 95% Confidence Interval 0.692–0.917 and asymptomatic locations ICC = 0.875; 95% Confidence Interval 0.759–0.935) [25].

### 2.5. Assessments: Muscle Stiffness

The procedure to obtain SWE was previously tested and showed excellent intra-examiner reliability (ICC > 0.95) and good and inter-examiner reliability (ICC > 0.8) [25]. Image acquisition and measurements were performed by a third single examiner with >10 years’ experience in musculoskeletal ultrasound and >5 years using SWE. Scans were obtained on a Canon Aplio A with a PLT-1005-BT 14L5 linear probe (5–14 MHz). Standardized console settings were used throughout (frequency 11 MHz, gain 78 dB, dynamic range 75 dB). Participants were positioned as during palpation and instructed to relax to avoid contraction-related morphological bias.

The transducer was placed over the marked landmarks for both MTrP and control sites with three priorities: (1) rotate to acquire a longitudinal view of the infraspinatus; (2) control anisotropy by aligning the beam parallel to muscle fibers; and (3) apply minimal pressure to prevent tissue deformation. With the probe maintained in position, three images per site were captured at ~5-s intervals. Each image was analyzed using a rectangular region of interest 1 cm in width and sufficiently tall to include the full muscle thickness while excluding superficial fascia and the underlying bone. The three SWE values per site were averaged for analysis and for assessing intra-rater reliability.

### 2.6. Statistical Analyses

Data processing and analyses were performed in IBM SPSS Statistics v29.1.1 for Mac OS (Armonk, NY, USA). All tests were two-tailed with α = 0.05. Distributional assumptions for continuous variables were inspected visually (histograms, Q–Q plots) and with Shapiro–Wilk tests. Descriptive statistics summarized participant characteristics and site-level measures (pressure pain threshold, PPT; shear-wave elastography, SWE). Normally distributed variables are reported as mean ± SD; otherwise as median.

Because each participant contributed a paired set of sites (latent MTrP vs. asymptomatic control) on the dominant side, within-subject differences in PPT and SWE were examined using paired Student’s *t*-tests.

Diagnostic accuracy was evaluated with receiver operating characteristic (ROC) analyses using site type (latent MTrP = positive; control = negative) as the criterion. Separate ROC curves were generated for PPT and SWE. The area under the ROC curve (AUC) with 95% confidence intervals quantified discrimination, with AUC ≥ 0.70 interpreted as acceptable performance [35]. The optimal cut-off for each marker was determined by maximizing Youden’s J (sensitivity + specificity − 1) [36]. At the selected cut-offs, we reported sensitivity, specificity, positive and negative likelihood ratios (LR+ and LR−), and their 95% CIs. Validity was deemed acceptable when sensitivity was ≥70% and specificity ≥50% [37]. Where relevant, AUCs for PPT and SWE were compared using DeLong’s test for correlated ROC curves. Directionality was handled so that lower PPT (greater mechanosensitivity) and higher SWE (greater stiffness) were coded to reflect higher probability of an MTrP; when necessary, variables were reversed for consistent ROC interpretation. In addition, precision–recall curves were plotted to display the relationship between positive predictive value (precision) and sensitivity (recall) across thresholds. The overall model quality metric corresponds to the area under the precision-recall curve (average precision), where higher values denote greater discriminative capacity and 0.5 reflects random classification.

## 3. Results

From a total of 80 individuals who were initially screened for participation, n = 4 participants were excluded because of presence of MTrPs. The remaining volunteers (n = 76, 63% females) met the eligibility criteria and were analyzed with no data losses. Regarding the demographic characteristics of the sample, participants had a mean age of 20.9 ± 4.2 years, a height of 1.72 ± 0.09, a weight of 65.2 ± 11.6 kg and a body mass index of 21.8 ± 2.7 kg/m^2^.

PPTs and muscle stiffness mean scores and differences between asymptomatic locations and latent MTrP are summarized in Table 1. The agreement between the three PPT measurements was good, with ICC = 0.81 (95% CI 0.66–0.90) for latent MTrPs and ICC = 0.86 (95% CI 0.74–0.93) for asymptomatic control sites. Latent infraspinatus MTrPs showed clear sensory and mechanical distinctions from adjacent asymptomatic tissue. Pressure algometry values were significantly lower at latent points than at control sites (49.7 ± 20.9 vs. 69.3 ± 30.8 N; mean difference 19.6 N, 95% CI 11.1–28.0; *p* < 0.001), indicating greater mechanosensitivity. SWE-derived stiffness measures also differentiated the sites: shear modulus was higher at latent points (34.8 ± 25.6 vs. 26.3 ± 11.7 kPa; mean difference 8.6 kPa, 95% CI 2.2–15.0; *p* = 0.009), and shear-wave speed was likewise increased (2.96 ± 1.17 vs. 2.62 ± 0.87 m/s; mean difference 0.34 m/s, 95% CI 0.01–0.67; *p* = 0.022). Although the stiffness metrics (particularly shear modulus) exhibited greater dispersion and therefore some overlap between conditions, the direction of effects was consistent across all measures. Expressed relative to control means, latent points demonstrated ~28% lower PPT, ~33% higher shear modulus, and ~13% higher shear-wave speed.

**Figure 2:**
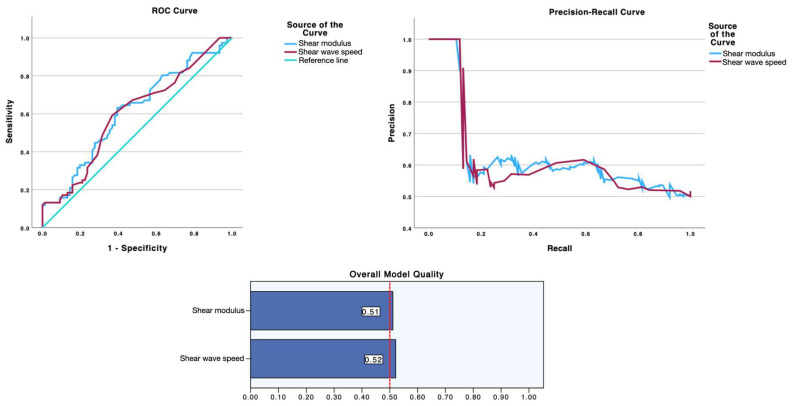
Discrimination of shear-wave elastography metrics for latent myofascial trigger points. Receiver operating characteristic (**left**) and precision–recall (**right**) curves for shear modulus (blue) and shear-wave speed (maroon), with the diagonal line indicating no discrimination and the dashed red line in the PR panel marking baseline precision (~0.50). Areas under the ROC curve were 0.611 for shear modulus and 0.601 for shear-wave speed (both *p* < 0.05). The “Overall Model Quality” bars (**bottom**) show values near 0.51–0.52, indicating only modest improvement over chance. Optimal cut-offs and operating characteristics are reported in Table 2.

**Figure 3:**
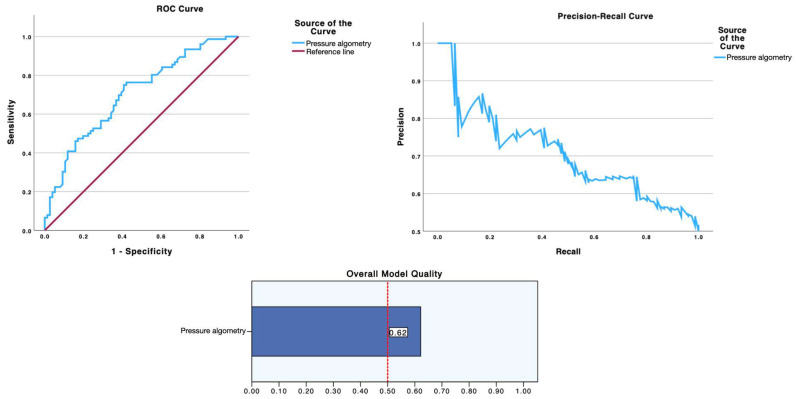
Diagnostic performance of pressure algometry for identifying latent myofascial trigger points. Receiver operating characteristic (**left**) and precision–recall (**right**) curves for pressure algometry (blue). The ROC AUC was 0.704 (95% CI 0.622–0.786; *p* < 0.001), exceeding the performance of the SWE metrics shown in Figure 1. The “Overall Model Quality” (**bottom**) is ~0.62, consistent with stronger precision across a broad recall range relative to SWE. The Youden-optimal cut-off was 47.5 N; corresponding sensitivity, specificity, and likelihood ratios are detailed in Table 2.

As summarized in Table 2 and visualized in Figure 1 (ROC curve, precision-recall curve and overall model quality for SWE) and Figure 2 (ROC curve, precision-recall curve, and overall model quality for pressure algometry), pressure algometry provided the most accurate single-marker discrimination between latent infraspinatus MTrPs and matched control sites. The AUC for algometry was 0.704 (95% CI 0.622–0.786; *p* < 0.001), exceeding the performance of both SWE metrics—shear modulus (AUC 0.611, 95% CI 0.521–0.701; *p* = 0.015) and shear-wave speed (AUC 0.601, 95% CI 0.511–0.692; *p* = 0.027). At the Youden-optimal cut-offs (47.5 N for algometry, 23.6 kPa for modulus, and 2.55 m/s for speed), sensitivity/specificity were 0.75/0.592, 0.632/0.605, and 0.592/0.632, respectively, yielding LR+ values of 1.84, 1.60, and 1.61, and LR− values of 0.42, 0.61, and 0.65. In practical terms, a positive algometry result produces a modest increase in the probability of a latent MTrP, while a negative algometry result provides the best rule-out value among the three measures. The graphical displays mirror these estimates: in ROC space (Figure 2 and Figure 3), the algometry curve lies farther from the diagonal than either SWE curve, and in the precision–recall panels the overall model quality is visibly higher for algometry (~0.62) than for SWE (~0.51–0.52), which hovers just above the baseline precision (≈0.50). Together, Table 2 and Figure 1 and Figure 2 indicate that algometry is the most informative adjunct for classifying latent MTrPs versus adjacent asymptomatic tissue, whereas SWE—though statistically significant—offers only small stand-alone diagnostic shifts and should be interpreted in conjunction with clinical criteria.

## 4. Discussion

Diagnosis has historically rested on careful palpation supplemented by characteristic history, but subjectivity and inter-examiner variability have limited consistency [38,39,40,41,42,43]. Earlier frameworks enumerated major features (spontaneous regional pain, referred pain, a taut band with a tender nodule, reduced range of motion) and minor features such as pain reproduction on pressure, a local twitch response, or relief after stretch or injection [13]. Because isolated signs vary in reliability, a multinational Delphi process proposed a pragmatic core cluster of three elements for identifying a trigger point [7,29]: a taut band, a hypersensitive spot, and referred pain, with the clinical separation of active and latent points based on whether palpation reproduces the patient’s familiar symptoms. The panel also clarified that referred phenomena may include a spectrum of sensations (deep ache, spreading pain, tingling, or burning) and cautioned against assuming fixed “X-mark” locations or rigid referral maps for each muscle. Reliability data suggest that identifying a taut band and a hypersensitive spot achieves moderate to near-perfect agreement in some settings, whereas the twitch response is poorly reliable and best treated as confirmatory rather than essential [38,39,40,41,42,43].

To the authors’ knowledge, this is the first report quantifying the diagnostic accuracy of pressure algometry and SWE to calculate the optimal cut-off point for differentiating latent MTrPs from asymptomatic locations within the infraspinatus muscle and providing sensitivity, specificity and positive/negative likehood ratio metrics. Diagnostic-accuracy designs are indispensable because all downstream treatment decisions hinge on whether a test correctly classifies patients; when diagnoses are wrong, even proven therapies can be ineffective or harmful. The distinction among active MTrPs, latent MTrPs and asymptomatic regions within muscles also carries practical implications. Active points are more likely to be the immediate drivers of a patient’s pain experience and functional limitation, often correlating with lower local pressure pain thresholds and broader sensory hypersensitivity [23]. Latent points, while not the primary pain generators at the time of examination, may act as load-sharing liabilities that impair movement quality and resilience; they can also serve as a reservoir from which active points emerge under stress, sleep deprivation, or sustained mechanical demand [44,45].

Indeed, discriminating latent MTrPs from truly asymptomatic muscle is clinically consequential rather than academic. Latent MTrPs can disturb motor recruitment, accelerate fatigue in agonists, and heighten antagonist activity, functioning as load-sharing liabilities and potential precursors of active, symptomatic foci [13]. Therefore, treating a latent infraspinatus MTrPs alongside its paired active point reduces pain and irritability of satellite MTrPs in the referred area, underscoring their system-level impact [23,44].

While discriminating active MTrPs from matched control regions is comparatively more straightforward (because, in addition to the biochemical milieu [46], symptom recognizability on palpation raises the pretest probability and typically yields larger between-site differences in mechanosensitivity [10]) the distinction between latent MTrPs and truly asymptomatic tissue is inherently more challenging. Latent MTrPs share the same palpable substrate (a hypersensitive spot within a taut band) yet do not reproduce the patient’s familiar symptoms, which results in smaller effect sizes, greater distributional overlap, and modest stand-alone likelihood ratios for objective adjuncts [47]. Consequently, establishing cut-points for objective adjuncts (e.g., PPT and SWE) with quantified sensitivity, specificity, and likelihood ratios against a Delphi-based reference is a necessary step to separate latent MTrPs from adjacent asymptomatic tissue in a way that is reproducible and clinically actionable.

Our results demonstrated that pressure algometry reached only acceptable accuracy, whereas SWE metrics were weaker, underscoring why explicit cut-offs and properly reported accuracy indices are needed beyond group-mean contrasts. This aligns with expert consensus that no single sign is pathognomonic and that MTrP identification should rely on a small cluster of findings rather than rigid maps, further justifying the present study’s focus on diagnostic-accuracy methodology. These results should be contrasted with a previous observational study assessing stiffness differences between latent MTrPs with asymptomatic control locations within the infraspinatus muscle and analyzing the correlation between PPTs and muscle stiffness [26]. In this study, latent MTrPs exhibited significantly lower PPTs than adjacent asymptomatic sites in both men and women, while SWE revealed no between-site or between-sex differences in resting muscle stiffness. Notably, greater stiffness correlated with higher PPTs (i.e., less mechanosensitivity), challenging the assumption that latent MTrPs are mechanically stiffer at rest. In contrast, our diagnostic-accuracy study, with a larger sample, replicated the PPT hypersensitivity and additionally detected small but statistically significant increases in SWE metrics at latent sites, yet these mechanical differences translated into only modest stand-alone discrimination compared with algometry.

Methodologically, both studies used identical acquisition protocols and blinding, so the divergent stiffness results likely reflect differences in statistical power (n = 40 vs. n = 76), analytic focus (group contrasts and correlations vs. ROC-based classification), and variance structure: a positive across-subject correlation between stiffness and PPT can coexist with within-subject site differences that are small in magnitude and easily diluted in smaller samples. Despite these differences and from a clinical perspective, both datasets converge on the same message: in the context of this study (with a sample of young and asymptomatic volunteers), PPT captures the core signal of latent MTrPs, while resting SWE changes are subtle and context-dependent, best interpreted as adjuncts rather than primary diagnostic criteria.

### Limitations

Several limitations should be acknowledged. This was a single-center study with a relatively young, convenience sample and was not powered for sex-stratified or side-specific analyses, which may limit generalizability. Latent MTrPs were identified by a single examiner (with no inter-examiner reliability data for MTrP diagnosis) and based on clinical criteria rather than a universally accepted gold standard, introducing potential misclassification and spectrum bias (especially because contrasts were restricted to latent sites versus asymptomatic controls, without active MTrPs or other shoulder pathologies). SWE acquisitions captured passive stiffness at rest using a single vendor and probe; despite strict standardization, SWE is sensitive to transducer pre-load, fiber orientation, ROI placement, and anisotropy, so residual measurement error and device-specific effects cannot be excluded and any proposed cut-offs may not transfer across systems or protocols. In addition, PPT and SWE thresholds and ROC estimates were derived and evaluated in the same dataset without external validation or calibration, increasing the risk of optimism; we did not explore multivariable models that combine mechanosensitivity with elastographic or clinical covariates. Finally, the cross-sectional design precludes inference about prognostic utility or treatment responsiveness; dynamic or contraction-dependent stiffness was not examined, and thresholds were not linked to clinically meaningful outcomes (gaps that future multi-center, pre-registered studies should address).

## 5. Conclusions

This study found that pressure algometry demonstrated acceptable ability to distinguish latent myofascial trigger points from adjacent asymptomatic infraspinatus tissue (AUC 0.704, optimal cut-off 47.5 N, sensitivity 0.75, specificity 0.592; LR+ 1.84, LR− 0.42), whereas shear-wave elastography, despite small but statistically significant between-site differences, showed only modest stand-alone discrimination (AUC 0.601–0.611). Taken together, these findings reinforce mechanosensitivity as an important measurable signal of latent MTrPs and position resting elastographic stiffness as an adjunct rather than a primary diagnostic criterion. Clinically, PPT may support standardized palpatory examination to improve classification of latent MTrPs, while SWE may add contextual information in equivocal cases or research settings. Future work should externally validate these cut-offs across devices and operators, explore multivariable models that combine mechanosensitivity with imaging or clinical covariates, and determine whether thresholds relate to prognosis or treatment response.

## Figures and Tables

**Table 1:** Pain pressure thresholds and shear wave elastography assessment for infraspinatus latent MTrPs and control locations.

	Pressure Algometry (N)	Muscle Stiffness	
		Shear Modulus (kPa)	Shear Wave Speed (m/s)
Latent Myofascial Trigger Point (n = 76)	49.7 ± 20.9	34.8 ± 25.6	2.96 ± 1.17
Control Point (n = 76)	69.3 ± 30.8	26.3 ± 11.7	2.62 ± 0.87
Difference Mean (95% Confidence Interval)	19.6 (11.1;28.0) *p* < 0.001	8.6 (2.2;15.0) *p* = 0.009	0.34 (0.01;0.67) *p* = 0.022
Cohen’s d	0.74 (0.42;1.06)	0.41 (0.10;0.71)	0.33 (0.02;0.63)

**Table 2:** Diagnostic accuracy analyses for pressure algometry and muscle stiffness in discriminating latent MTrP and asymptomatic muscle locations.

	Pressure Algometry	Muscle stiffness	
		Shear Modulus	Shear Wave Speed
ROC Value	0.70	0.61	0.60
95% Confidence Interval	0.62–0.79	0.52–0.70	0.51–0.69
Optimal cut-off	47.5	23.6	2.55
Significance	<0.001	0.015	0.027
Sensitivity	0.75 (0.64;0.84)	0.63 (0.51;0.74)	0.59 (0.47;0.70)
1 − Specificity	0.59 (0.48;0.69)	0.61 (0.49;0.71)	0.63 (0.51;0.73)
Youden Index	0.34	0.24	0.22
LR+	1.84 (1.36;2.49)	1.60 (1.17;2.18)	1.61 (1.17;2.23)
LR−	0.42 (0.29;0.61)	0.61 (0.44;0.85)	0.65 (0.48;0.88)

## Data Availability

The raw data supporting the conclusions of this article will be made available by the authors on request.
